# Extended Use of McGregor Pedicled Groin Flap for Forearm: A Case Series

**DOI:** 10.7759/cureus.42375

**Published:** 2023-07-24

**Authors:** Mayank Tripathi, Soumitra Saha, Kumar Vineet, Pavithira GJ, Shraddha Modi

**Affiliations:** 1 Surgical Oncology, Mahamana Pandit Madan Mohan Malaviya Cancer Centre (MPMMCC) & Homi Bhabha Cancer Hospital (HBCH), Varanasi, IND; 2 Pediatric Surgical Oncology, Mahamana Pandit Madan Mohan Malaviya Cancer Centre (MPMMCC) & Homi Bhabha Cancer Hospital (HBCH), Varanasi, IND

**Keywords:** hand, forearm, flap, pedicled, mcgregor

## Abstract

Tissue loss in the forearm is a common entity. There are various etiologies to tissue loss like trauma, burn, post-surgery, etc. Coverage of defect or tissue loss of the forearm is a surgical challenge and options available for forearm coverage are limited. To bypass this difficulty, McGregor pedicled groin flap is used by us to cover large forearm defects with successful outcomes in this case series.

## Introduction

McGregor flap is a pedicled flap from the groin region. It is a type of axial flap based on the superficial circumflex iliac artery. It is a reliable flap for coverage of tissue defects in the forearm up to the elbow [1]. Coverage of skin or tissue loss of the forearm is a surgical challenge. Groin flap and random abdominal flaps are standard techniques for the reconstruction of forearm defects [2]. Even though a free flap is an excellent option in such cases, the main barrier to such reconstruction is logistics and expertise, therefore the groin flap remains a valuable technique for upper limb defects. We have even used it with success for more proximal defects around the elbow.

## Case presentation

We here present a case series of four cases where McGregor's pedicled groin flap has been used for forearm defects and elbow defects. Three of the four cases had a forearm and one patient had a defect around the elbow (Table 1).

**Table 1:** Details of cases in which McGregor flap was applied

	Diagnosis	Anatomical site of defect	Defect size
Case -1	Ewing’s sarcoma	Forearm	12x7 cm
Case-2	Rhabdomyosarcoma	Forearm	10x8 cm
Case -3	Dermatofibrosarcoma protuberans	Forearm	9x6 cm
Case 4	Squamous cell carcinoma	Dorsum of forearm around elbow joint	9x5 cm

Case one

An 18-year-old adolescent presented to our side with complaints of pain and swelling in the forearm for a month. On examination, there was 9x8 cm swelling on the ulnar side of the right forearm. On biopsy of the swelling, it came out as Ewing’s sarcoma. There was no evidence of metastasis on the workup. The patient received chemotherapy followed by wide local excision with McGregor pedicled groin flap. The patient was followed up every three months for two years. 

Case two

**Figure 1:**
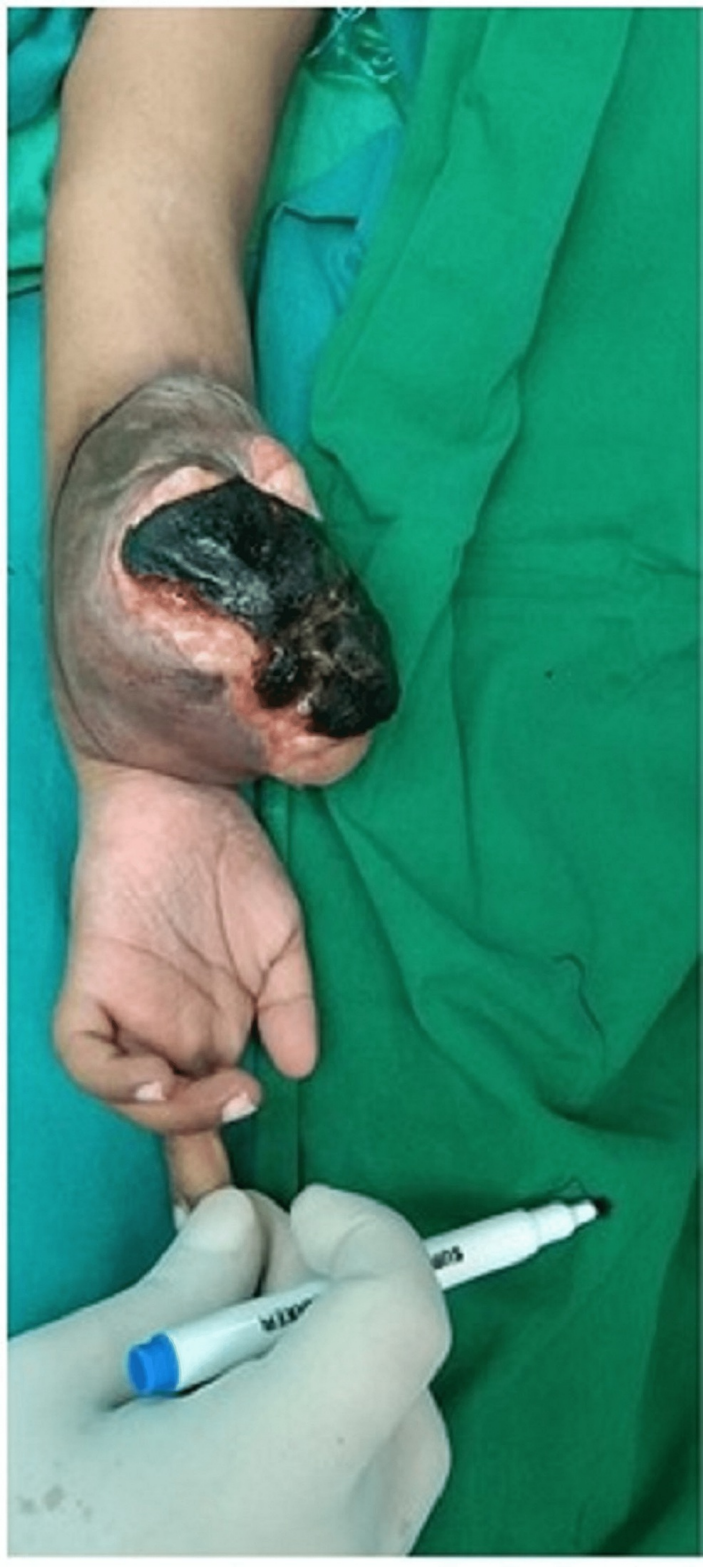
Fungating mass in left forearm- Preoperative image

**Figure 2:**
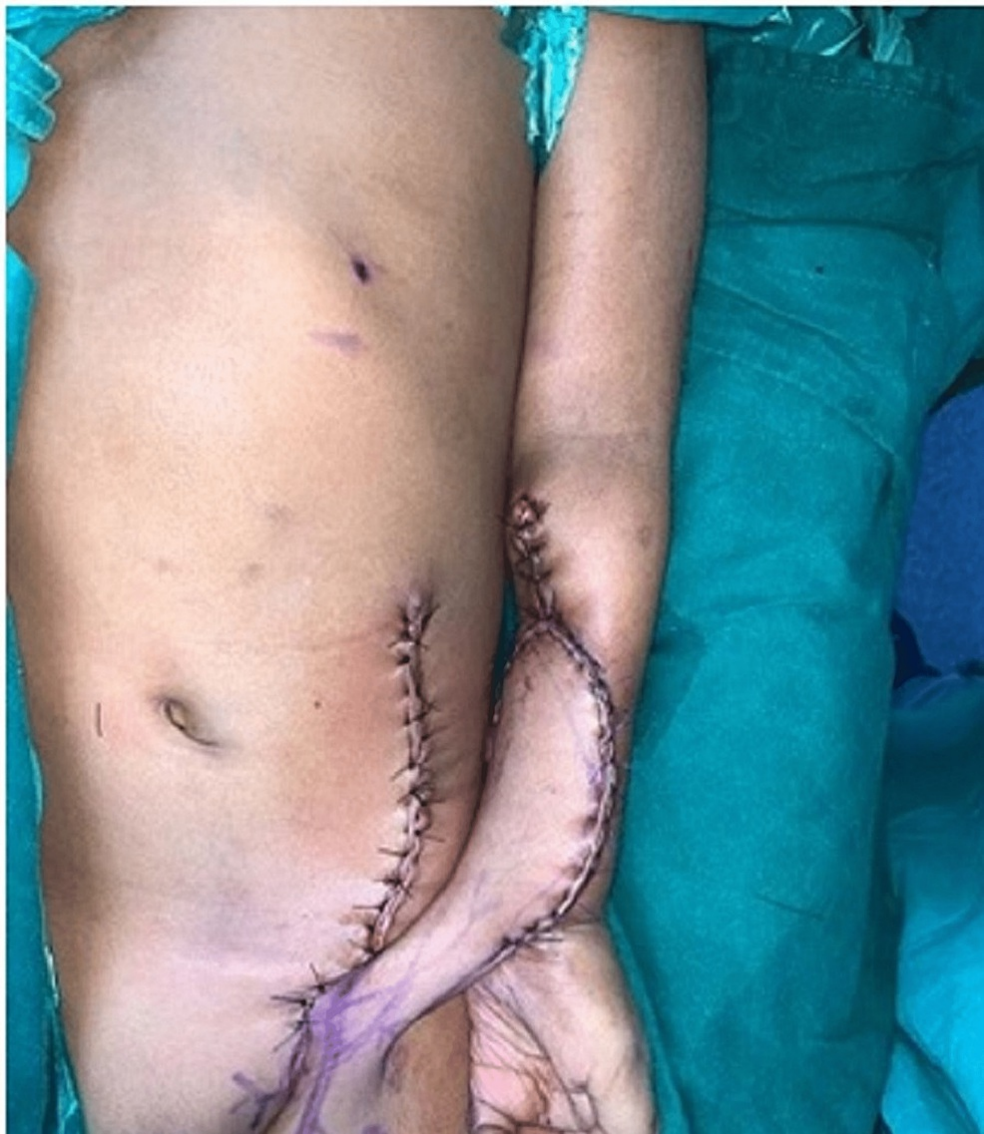
McGregor pedicled groin flap- Intraoperative image

**Figure 3:**
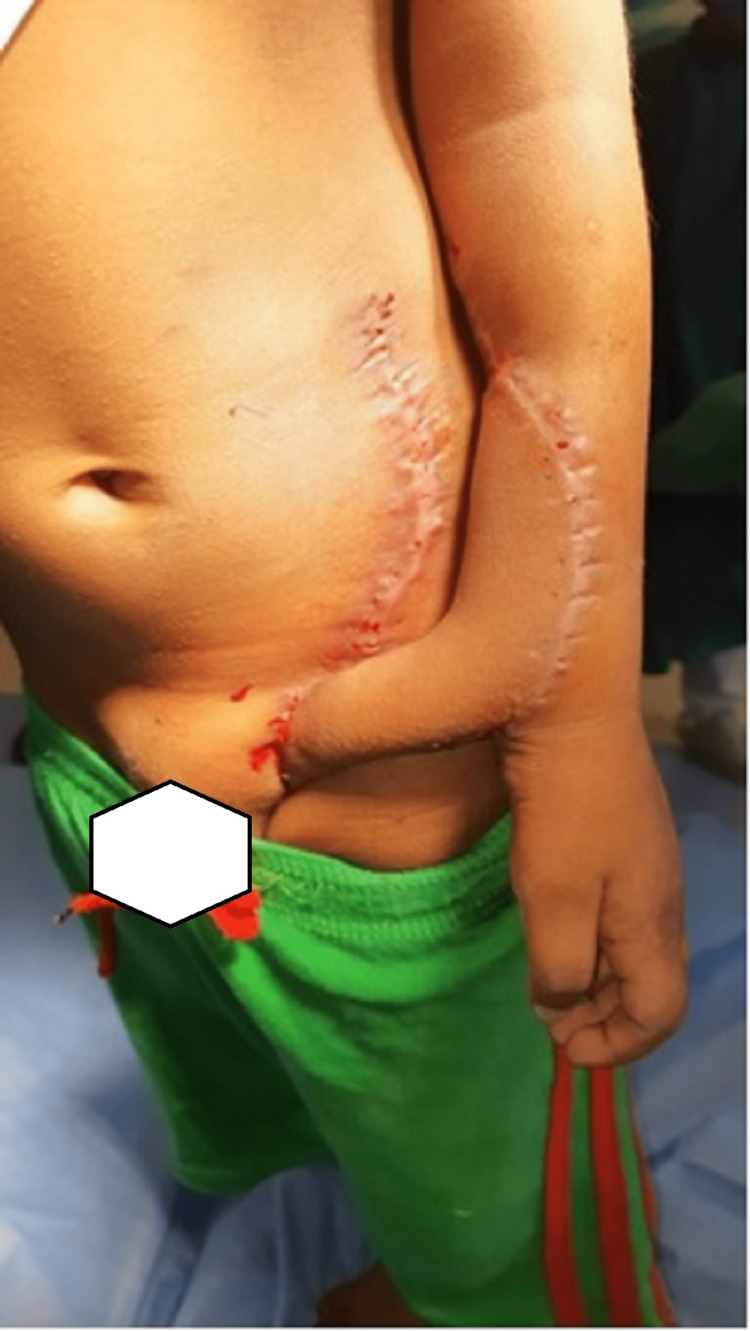
Postoperative image of McGregor pedicled groin flap

A four-year-old boy came to our outdoor services with a complaint of swelling and pain over his left forearm for two months. The swelling was 8x7 cm large with an associated 4x5 cm fungating mass (Figure 1). The preoperative biopsy result was suggestive of round-cell sarcoma. There was no evidence of metastasis on the workup. The patient received neoadjuvant chemotherapy and underwent wide local excision of the anterior compartment of the left forearm with McGregor pedicled groin flap (Figures 2,3). The post-operative histopathological report suggested rhabdomyosarcoma. He received adjuvant concurrent chemoradiotherapy and is currently disease free. The patient is on follow-up for the last two and a half years. 

Case three

A 46-year-old gentleman presented to us with dermatofibrosarcoma protuberans of the left forearm. He was evaluated outside and was diagnosed with the above condition. Mass was 7x5 cm in size with no metastasis. The patient underwent local excision of the mass with McGregor's pedicled groin flap. The post-operative period was uneventful and the patient is currently doing well. He is on yearly follow-up and was last followed up six months back.

Case four

**Figure 4:**
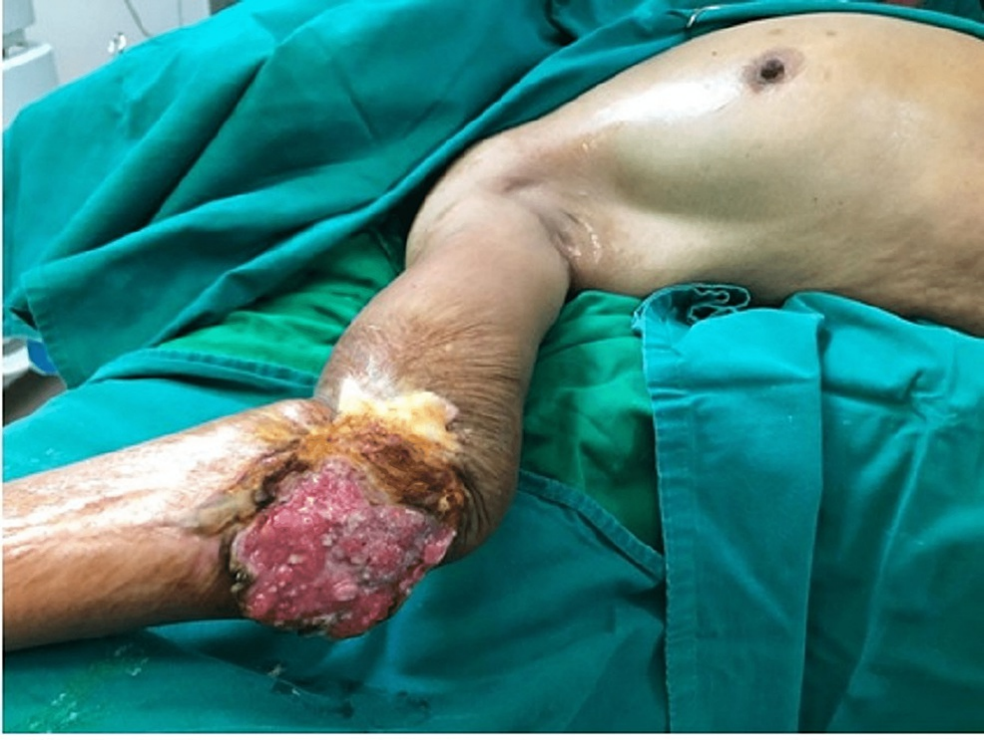
Non-healing ulcer over right elbow- preoperative image

**Figure 5:**
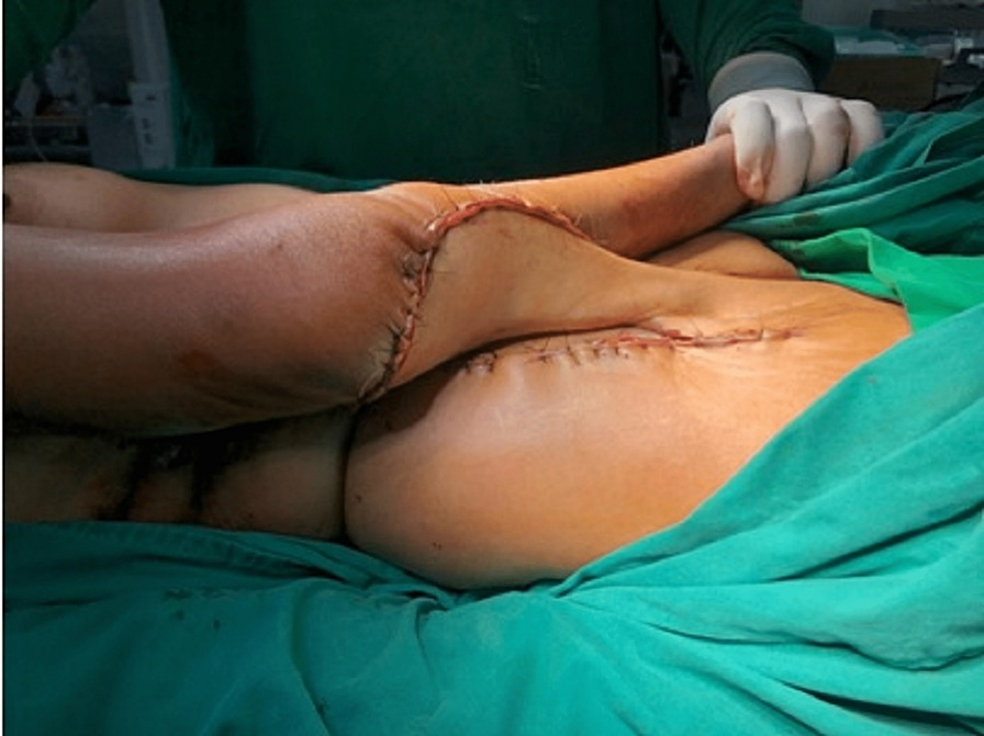
McGregor pedicled groin flap- Intraoperative image

A 54-year-old lady presented to our side with repeated non-healing ulcers over her right elbow for seven years (Figure 4). She had burn injuries over her right elbow and forearm 20 years back. The biopsy result of non-healing ulcer was suggestive of squamous cell carcinoma. She was diagnosed with Marjolin's ulcer. On metastatic workup, no evidence of metastasis was seen. The patient underwent wide local excision of Marjolin’s ulcer with a McGregor pedicled groin flap (Figure 5). The post-operative histopathological report suggested moderately differentiated squamous cell carcinoma with tumor size 7x7x1.2 cm. The postoperative period was uneventful and she is under follow-up from an oncological point of view for the last two years. 

Surgical technique

After wide local excision of the tumor and confirmation of free margins on the frozen section, the pedicled groin flap was raised as follows. Two important bony landmarks for the pedicled groin flap are the anterior superior iliac spine (ASIS) and pubic tubercle. These two points are joined by a line with a convexity representing the course of the inguinal ligament. The femoral artery is palpated and its course is marked in the inguinal area. A point is marked 2.5 cm lateral to the femoral artery and 2.5 cm below the inguinal ligament. This point represents an area where the superficial circumflex artery becomes subcutaneous hence while raising the flap, we should not go medial to this point else the pedicle will be at risk.

**Figure 6:**
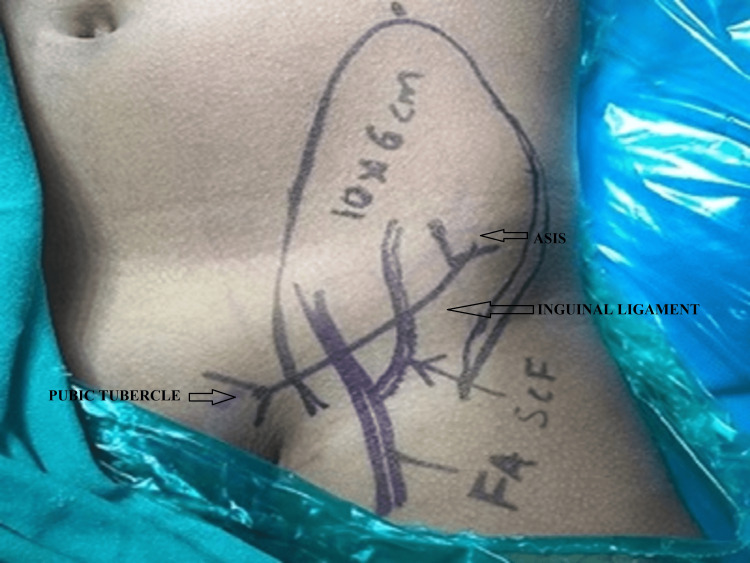
Surface Landmarks for groin flap- Landmarks in the above image are ASIS, pubic tubercle ,inguinal ligament and femoral artery ASIS- Anterior Superior Iliac Spine, FA- Femoral artery, SCF- Superficial Circumflex iliac artery from Femoral artery

The course of the superficial circumflex iliac artery is marked as it crosses the inguinal ligament in its lateral third. Further, after crossing the inguinal ligament, it courses more or less in a straight line. The required flap is marked keeping in mind that the marked flap is around 2/3rd below the inguinal ligament which is the axial portion of the flap, the area above the inguinal ligament represents the random part of the groin flap. (Figure 6). 

However, in practice flap size can be equally distributed both above and below the inguinal ligament. The flap size can be extended till the mid-axillary line on the lateral side. If we want to raise a longer flap, the width of the flap should be more to incorporate more perforators. For a better arc of rotation, the base can be narrowed in the axial portion i.e. below the inguinal ligament.

We started raising the flap from the abdominal portion staying above the external oblique aponeurosis and gradually proceeded towards the inguinal ligament. The origin of the superficial circumflex iliac artery was identified after palpating the common femoral artery. Flap was raised from lateral to medial direction in the part lying below the inguinal ligament till the point where the superficial circumflex iliac artery becomes subcutaneous. Donor site defects are usually closed primarily, larger defects may need skin grafts. Flexing the hip may aid in the primary closure of a large donor site. Further, a hand was brought into the inguinal region and a flap was adapted to the defect. The posterior edge of the flap was sutured before the anterior edge. At last paraffin wax dressing was applied on all suture sites. After the inset of the flap to the defect we practice restraining the upper limb in the initial four days using bandages and the patient is taught to keep the upper limb in a position that causes minimum traction on the pedicle. Both of our younger patients learned the position in a day and older ones took three to four days for the same. Preoperative education regarding non-mobilization of the involved upper limb for three weeks was done and agreement from the patient and their caregivers was taken for the same. If the patient is not ready for the postoperative limb position, this flap should not be done. Flap divisions in three cases were done at three weeks however for the defect around the elbow it was done at two weeks to avoid shoulder dysfunction. Flap division was performed under local anesthesia in all four cases.

Results

There were no flap-related complications in all four cases. Cosmetic outcomes were acceptable. Recipient site paraesthesia was the only complaint these patients had on the first follow-up after three months. None of the patients had donor site complications.

## Discussion

Groin flap is an axial pattern flap based on the superficial circumflex iliac artery, the anterolateral branch of the femoral artery, and the superficial venous network of the groin area. The superficial circumflex artery in about 50% of cases has a common trunk with the superficial inferior epigastric artery [3]. As per Cormack and Lamberty's classification (6 C’s) [4], the blood supply of the flap comes from a direct cutaneous artery i.e. superficial circumflex iliac artery and it consists of skin and subcutaneous tissue (cutaneous flap). 

This flap was first described by McGregor and Jackson as a pedicled skin flap following their work on deltopectoral flap and autonomous vascularisation where arteries and veins are close to their origins and termination and was later popularized by Knutson for various hand defects. O’Brien et al. [5] used this flap as a free flap which was later modified by Acland as a free iliac flap with a lateral skin island [6]. 

In their study with 49 patients for pedicled groin flap for reconstruction of hand defect, Goertz et al. claimed that finger, thumb, and dorsal hand have shown the best results with groin flap whereas forearm and palm procedure has been less successful mainly because of their large area [7]. 

It can be used as both a local and distant flap (contiguity). Vascular flow in the flap is antegrade. Elevation of the flap can be delayed and a tissue expander can also be used to harvest a larger flap. The flap can be tubed especially for hand defects and can also be used as a thin pliable cover (conformation). The long pedicle of the groin flap has several advantages, mainly lesser pre-operative planning, and can be done by inexperienced surgeons. It can even be used in many emergency settings with minimal infection risk. Moreover, the relative constancy of the vascular pedicle even in anatomical position made this flap popular [1].

Besides the advantages of this flap, the major side effect of the groin flap is shoulder stiffness due to the uncomfortable position of the arm for a long time in the postoperative period which can be treated easily with early physiotherapy. In our case report, the groin flap has given an excellent tissue cover for the forearm defects with good cosmetic and functional outcomes as there is easy concealment of donor scar. In some cases, the groin flap can be thicker than the forearm skin and if it bothers the patient, liposuction can be done in the future to decrease flap thickness [8]. 

## Conclusions

We recommend the use of a groin flap for forearm and hand defects especially in resource-constrained settings as it fulfills the criteria and enables a remote autoplasty. It also allows the coverage of large and complex soft tissue defects of the hand and forearm in other conditions like wounds, traumatic amputations, degloving injury, burns, burn scars release, and including tumor excision, especially when noble structures (tendons, nerves, and vessels) are exposed.
